# Psychopathic traits and offender characteristics – a nationwide consecutive sample of homicidal male adolescents

**DOI:** 10.1186/1471-244X-9-18

**Published:** 2009-05-06

**Authors:** Nina Lindberg, Taina Laajasalo, Matti Holi, Hanna Putkonen, Ghitta Weizmann-Henelius, Helinä Häkkänen-Nyholm

**Affiliations:** 1 Helsinki University Central Hospital, Department of Adolescent Psychiatry, PO Box 590, 00029 HUS/HYKS, Helsinki, Finland; 2 Department of Psychology, PO Box 9, 00014 University of Helsinki, Finland; 3 Kellokoski Hospital, 04500 Kellokoski, Finland; 4 Vanha Vaasa Hospital, PO Box 13, 65381 Vaasa, Finland; 5 Forensic Laboratory, National Bureau of Investigation, PO Box 285, 01301 Vantaa, Finland

## Abstract

**Background:**

The aim of the study was to evaluate psychopathy-like personality traits in a nationwide consecutive sample of adolescent male homicide offenders and to compare the findings with those of a randomly sampled adult male homicide offender group. A further aim was to investigate associations between psychopathic traits and offender and offence characteristics in adolescent homicides.

**Methods:**

Forensic psychiatric examination reports and crime reports of all 15 to19- year- old male Finnish offenders who had been subjected to a forensic psychiatric examination and convicted for a homicide during 1995–2004 were collected (n = 57). A random sample of 57 adult male homicide offenders was selected as a comparison group. Offence and offender characteristics were collected from the files and a file-based assessment of psychopathic traits was performed using the Hare Psychopathy Checklist-Revised (PCL-R) by trained raters.

**Results:**

No significant differences existed between the adolescents and adults in PCL-R total scores, factor 2 (social deviance) scores, or in facets 3 (lifestyle) and 4 (antisocial). Adults scored significantly higher on factor 1 (interpersonal/affective) and facets 1 (interpersonal) and 2 (affective). The adolescent group was divided into two subgroups according to PCL-R total scores. One in five homicidal male adolescents met criteria for psychopathic personality using a PCL-R total score of 26 or higher. These boys significantly more often had a crime history before the index homicide, more frequently used excessive violence during the index homicide, more rarely lived with both parents until 16 years of age, had more institutional or foster home placements in childhood, had more school difficulties, more often had received special education, and, more often had contact with mental health services prior to age 18 years than boys scoring low on the PCL-R. They also more often had parental criminal history as well as homicide history of parents or near relatives than the group scoring low on the PCL-R.

**Conclusion:**

Homicidal boys behaved as antisocially as the homicidal adults. The adults, however, showed more both affective and interpersonal features of psychopathy. Homicidal adolescents with psychopathy-like personality character form a special subgroup among other homicidal youngsters. Recognizing their characteristics, especially in life course development, would facilitate effective prevention and intervention efforts.

## Background

Among Western European nations, Finland has an exceptionally high rate of homicide. In 2006, the total rate per 100,000 inhabitants of homicidal crimes reported to the police was 2.6 [1] in a population of 5.3 million [2]. The annual number of police-reported homicides has varied between 100 and 155 during the last ten years [3]. The rates per capita have for decades been about double the rate of the most of the other West European democracies and triple the rate of the other Nordic countries [4]. Approximately 9% of homicides each year are committed by individuals aged less than 20 years [5]. As many as 92% of the young homicidal offenders are boys [6].

According to a recent study on Finnish homicidal adolescents [7], approximately 50% were diagnosed as having a conduct disorder or a personality disorder, while 7% of these offenders suffered from schizophrenia. Sixty-four percent of the adolescents were intoxicated by alcohol and 21% were under the influence of drugs at the time of the killing; however, as many as 32% of the offenders were considered not to suffer from a mental illness or substance abuse. The motive "robbery" was high [7] suggesting that among adolescent homicide offenders acts of instrumental violence are more frequent. About one-third of homicides committed by adolescents are carried out by two or more perpetrators [6]. Excessive violence has been shown to be related to adolescent homicides, suggesting an unambivalent need to maximize injuries [8] or a tendency to show off to peers [7].

Psychopathy is defined as a constellation of affective, interpersonal, and behavioral characteristics including impulsivity, irresponsibility, shallow emotions, lack of empathy, guilt, or remorse, pathological lying, and persistent violation of social norms and expectations [9-11]. At the interpersonal level, psychopathic individuals have been described as grandiose, arrogant, callous, dominant, superficial, and manipulative. Affectively, they are short-tempered, unable to form strong emotional bonds with others, and lacking in guilt or anxiety. These interpersonal and affective features are associated with a socially deviant lifestyle that includes irresponsible behavior and a tendency to ignore or violate social conventions and mores [10]. Although not all persons with psychopathy come into contact with the criminal system, their defining features place them at high risk for aggression and violence [12]. Offenders with psychopathy typically begin their antisocial and criminal activities at a relatively young age and continue to engage in these activities throughout their lifespan [13]. In addition, their use of violence tends to be more instrumental, dispassionate, and predatory than that of other offenders [14]. Psychopathy has also been repeatedly associated with sadistic and sexual violence [15]. Psychopathic criminals re-offend more quickly, more often, and more violently following release from custody than do other offenders [16]. Victims of offenders with psychopathy are less often family members and more often strangers than is the case with other nonpsychopathic violent offenders [17]. In addition to antisocial personality disorder, psychopathy is associated with alcohol and drug abuse and dependency [18,19]. Usually, a negative correlation or no correlation with the presence of a major mental disorder is recorded [18,20]. However, prevalence of psychopathy among seriously violent offenders with schizophrenia is elevated, being as high as 20% [21-23].

Most authors consider adult psychopathy to stem from conduct problems exhibited earlier in life [24], and childhood traumatization has repeatedly been associated with psychopathic development [25-27], but the utility of the psychopathy construct has been questioned for youths by some researchers because of its likelihood to be less stable, the weight that the label carries for poor prognosis, and the lack of prospective longitudinal research [28-30]. A recent follow-up study has, however, found that adolescent psychopathic features are quite stable [31], and depending on the nature of the sample and the instrument used to assess psychopathy, as many as 9–59% of adolescent offenders have been reported to have psychopathy- like personality character [26]. Youngsters with psychopathic traits commit more violent acts [32], re-offend more quickly than other antisocial adolescents [33], and their offences are more serious than in other antisocial youths [34]. So, in this sense, juvenile psychopathy resembles adult psychopathy [35].

The aim of this study was to evaluate psychopathy-like personality traits in a nationwide consecutive sample of adolescent male homicide offenders and to compare these findings with a randomly sampled adult gender-matched homicide offender group. Our hypothesis was that despite the difference in age, in the perspective of psychopathy these two groups would not differ from each other. The other aim of the study was to investigate associations between adolescent psychopathic traits and offender and offence characteristics. Our hypotheses were that adolescents with psychopathy-like personality character would more often have previous crime history, more often suffer from conduct disorder/antisocial personality disorder and drug problems, significantly more frequently use excessive violence in their killing, more often act with co-offenders, more often direct violence to strangers, and, that motive for violence would more often be instrumental. We also examined connections between adolescent psychopathic traits and life course development. We hypothesised that homicidal adolescents with psychopathic personality character would report more childhood trauma.

## Methods

### Sample

The material of the present study was register-based and nationwide. In Finland, the mean clearance rate for homicide was 92% during 1995–2004 [2]. Information concerning homicides and the offenders was obtained from the Finnish National Authority for Medicolegal Affairs (NAMA), which organizes the forensic psychiatric examinations in Finland. According to Finnish law, courts decide whether a forensic examination is needed. After deciding on the examination, the court asks NAMA to arrange it. Forensic psychiatric examinations are inpatient evaluations lasting six weeks on average, and include data gathered from various sources (family members, relatives, and medical, criminal, school, and military records), psychiatric evaluation, standardized psychological tests, interviews conducted by a multiprofessional team, evaluation of the offender's physical condition and continuous observation of the offender by hospital staff. The final forensic psychiatric report includes an opinion on the level of criminal responsibility, a possible psychiatric diagnosis, and an assessment as to whether or not the offender fulfils the criteria for involuntary psychiatric care. As part of a large research project on Finnish homicides led by author HH, forensic psychiatric examination reports of all offenders prosecuted for a homicide perpetrated in 1995–2004 and who had been subjected to a forensic psychiatric examination were collected from the archives of NAMA. Between 1995–2004 a total of 1046 people were charged with homicide offences [2], 749 of whom were referred to a forensic psychiatric examination. These 749 offenders were prosecuted for 700 homicidal events with a total of 757 victims. Of these offenders, 66 (9%) were 15–19-years old at the time of the killing (note: in Finland, the minimum age of criminal liability is 15 years). Later, collection of subjects' criminal records from the Legal Register Center showed that six of these 15- to 19- year-olds were eventually not convicted for the homicide (but rather for aggravated assault, for instance) or did not have a criminal record (e.g. due to being deceased). These cases were excluded from the data leaving us with a sample of 57 boys and 3 girls. After excluding the girls (due to an extremely small number) the final data comprised 57 male adolescents (mean age 17.6 years, SD 1.25). Of the remaining offenders in the national data, a random sample of 57 adult males (mean age 37.6 years, range 20–59, SD 10.81) convicted of a homicide served as the comparison group. NAMA, the Legal Register Center and the Ministry of the Interior approved the study protocol.

### Measures

Assessment of psychopathy-like personality character was performed using the 20-item Hare Psychopathy Checklist-Revised (PCL-R) [10], which has become the standard for assessing psychopathy in forensic settings. The PCL-R is thus a reliable and valid instrument for measuring psychopathy [36-40], and its psychometric properties appear to be much the same across countries [39]. Although the PCL-R was originally constructed for use with adult male criminal offenders, it has been shown to be functional in the assessment of male juvenile delinquents as well [41,42]. Due to the comparison with an adult sample, the youth version of PCL-R (PCL-R:YV) [43] was not applicable here. Specific scoring criteria were used to rate each PCL-R item on a three-point scale (0 = absent,1 = possibly or partially present, 2 = definitely present) according to the extent to which it applies to a given individual. The PCL-R items are summed to yield total scores ranging from zero to 40; scores of 30 and higher are considered diagnostic of psychopathy [44]. In line with recommendations of a lower cut-off score for European populations [40,45,46], a cut-off score of 26 has often been used in studies performed in Scandinavian countries [47,48]. The PCL-R is underpinned by two factors that tap affective-interpersonal features (factor 1: glibness and superficial charm, grandiose sense of self-worth, pathological lying, manipulative behavior, lack of remorse or guilt, shallow affect, lack of empathy, failure to accept responsibility) and socially deviant lifestyle and behaviors (factor 2: proneness to boredom, parasitic lifestyle, poor behavioral controls, lack of realistic, long-term goals, impulsivity, irresponsibility, juvenile delinquency, revocation of conditional release). Factor 1 can be separated into two facets; interpersonal (facet 1) and affective (facet 2), as can factor 2; lifestyle (facet 3) and antisocial (facet 4). Although PCL-R assessments are recommended to be based on both a review of file information and an semistructured interview with the offender, research has consistently shown that assessments based solely on file information are highly similar to those including an interview, and, provided that there is sufficient file information, are appropriate in the absence of an interview, especially for research purposes [36,49-51].

### Procedure

Forensic psychiatric examination reports were retrospectively reviewed. As PCL-R/PCL-R:YV is not applied in the standard hospital examinations, the reports were retrospectively scored using the PCL-R by trained raters, all of whom were either forensic psychiatrists or psychologists. Further, to evaluate inter-rater agreement on PCL-R ratings, 20 reports were randomly chosen from the total national data and rated by all raters after workshop attendance and several training sessions. The inter-rater agreement was assessed using intraclass correlation (ICC). The ICC was 0.898 for PCL-R total score, 0.735 for factor 1 score and 0.920 for factor 2 score. All correlations were significant (p < 0.001). The internal consistency, as measured by Cronbach's alpha, was 0.89 for all items, 0.86 for factor 1, and 0.79 for factor 2, 0.84 for facet 1, 0.83 for facet 2, 0.87 for facet 3, and 0.64 for facet 4.

Demographic data, family related characteristics, problems related to school, information on psychiatric and criminal history as well as the index offences were gathered from the Finnish police computerized Criminal Index File and the forensic psychiatric evaluation reports. Diagnoses were made according to DSM-III-R [52] criteria until 1996, after which ICD-10 [53] was used together with DSM-IV [54]. According to Cloninger and Svrakic [55] diagnosis of specific personality disorder may be made in children and adolescents when observed maladaptive personality traits are pervasive, persistent, and unlikely to be limited to a particular developmental stage or an episode of an axis I disorder. Diagnosis of a personality disorder in an individual under 18 years of age requires that the features be present for more than 1 year. The only exception to this is antisocial personality disorder, which can not be diagnosed in individuals under 18 years of age. The overall quality and reliability of Finnish forensic psyhiatric examinations are considered high by both courts and scientists [56]. With regard to index violence, the cases needed to be coded for excessive violence. There is currently no uniform operational definition for excessive violence. However, excessive violence has been operationalised in two of our previous studies on homicides in reference to the mean number of stab wounds in the data as well as sadistic or sexual features [7,57]. Similar operational definition was used in the present study to allow comparison to our previous studies. Thus, with regard to index violence, the cases were classified as excessively violent if sadistic or sexual features, mutilation, more than three forms of violence, or more than 13 stab wounds (which was the mean number of stab wounds with a s.d. of 23.4) were present. Inter-rater reliability of the offence, victim, and offender related variables has been assessed in our previous studies, where the same data collection procedure and partly the same data were used [58,59]. Thus, only variables with substantial or perfect agreement [60] were included in this data.

### Statistics

Data analyses were conducted with the SPSS 11.0.1. statistical software package. Chi-square analysis and Fisher's Exact Test were used to compare differences in frequencies between the groups. Differences in mean PCL-R scores were assessed by Mann-Whitney U-test. Findings were considered significant when p < 0.05. The Bonferroni correction was not used to control Type I errors due to the multiple comparisons, as it has been criticized for dramatically increasing the risk of Type II errors [61-63]. Instead, effect sizes are reported. For chi square analysis, the magnitudes of effect size phi were interpreted following the guidelines by Rea and Parker: 0.00 to under 0.10 – negligible association, 0.10 to under 0.20 – weak association, 0.20 to under 0.40 – moderate association, 0.40 to under 0.60 – relatively strong association, 0.60 to under 0.80 – strong association, and 0.80 to 1.00 – very strong association [64]. To assist in determining the meaningfulness of group effects, correlational effect size statistics were calculated by diving the z score by the square root of the number of participants contributing to the analyses. An effect size of r = 0.10 was defined as small, r = 0.30 as medium, and r = 0.50 as large [65].

## Results

The mean age of victims of the adolescent group was 38.3 years (range 10–78, SD 19.01) and of the adult comparison group 40.3 years (range 3–85, SD 13.65). The difference between the groups was not significant.

The PCL-R total scores, factor scores, facet scores, and item-by-item scores are presented in Table 1. Because of the young age of the adolescents, the item " many short-term marital relationships" was excluded from the analyses.

**Table 1:** PCL-R mean (SD) total scores, factor scores, facet scores, and item-by-item scores of the homicidal male adolescents (n = 57) and the adult male comparison group (n = 57).

	Adolescents	Comparisons	Statistics	p	Phi
PCL-R total score	18.1 (8.73)	20.2 (10.79)	-1.083	0.279	-0.10
PCL-R total score ≥ 26 n (%)	12/57 (21)	21/57 (37)	3.455	0.063	-0.17
PCL-R total score > 30 n (%)	7/57 (12)	17/57 (30)	5.278	0.022	-0.22
PCL-R factor 1 Interpersonal/Affective	5.5 (3.55)	8.3 (4.88)	-3.090	0.002	-0.29
PCL-R factor 2 Social Deviance	11.4 (5.50)	10.5 (6.08)	-0.675	0.500	-0.06
PCL-R facet 1 Interpersonal	1.29 (1.70)	2.80 (2.76)	-3.115	0.002	-0.30
PCL-R facet 2 Affective	4.52 (2.80)	5.50 (2.66)	-2.475	0.013	-0.23
PCL-R facet 3 Lifestyle	6.48 (3.48)	6.05 (3.50)	-0.824	0.410	-0.07
PCL-R facet 4 Antisocial	5.16 (2.68)	4.78 (3.13)	-0.811	0.417	-0.08
					
1. Glibness/superficial charm	0.3 (0.43)	0.5 (0.76)	-1.143	0.253	-0.11
2. Grandiose sense of self-worth	0.4 (0.65)	0.8 (0.86)	-2.472	0.013	-0.23
3. Need for stimulation	1.3 (0.81)	1.3 (0.81)	-0.638	0.524	-0.05
4. Pathological lying	0.1 (0.47)	0.6 (0.83)	-4.019	0.000	-0.38
5. Conning/manipulative	0.5 (0.74)	0.8 (0.88)	-2.060	0.039	-0.20
6. Lack of remorse or guilt	1.2 (0.81)	1.3 (0.87)	0.830	0.407	-0.08
7. Shallow affect	0.7 (0.76)	1.5 (0.63)	-5.566	0.000	-0.52
8. Callous/lack of empathy	1.3 (1.49)	1.4 (0.78)	-1.614	0.107	-0.15
9. Parasitic lifestyle	0.9 (0.84)	0.9 (0.81)	-0.216	0.829	-0.02
10. Poor behavioral controls	1.3 (0.76)	1.5 (0.71)	-1.779	0.075	-0.17
11. Promiscuous sexual behaviour	0.3 (0.63)	0.4 (0.67)	-0.696	0.487	-0.07
12. Early behavioral problems	0.8 (0.88)	0.7 (0.82)	-0.831	0.406	-0.08
13. Lack of realistic goals	1.3 (0.86)	1.2 (0.82)	-0.434	0.664	-0.04
14. Impulsivity	1.7 (0.55)	1.4 (0.78)	-1.224	0.221	-0.11
15. Irresponsibility	1.3 (0.83)	1.3 (0.85)	-0.487	0.626	-0.05
16. Failure to accept responsibility	1.4 (1.48)	1.3 (0.82)	-0.869	0.385	0.08
17. Many short-term marital relationships		0.3 (0.64)			
18. Juvenile delinquency	1.6 (0.76)	0.7 (0.92)	-4.803	0.000	-0.46
19. Revocation of conditional release	0.8 (0.98)	0.8 (0.97)	-0.140	0.889	-0.02
20. Criminal versatility	0.7 (0.83)	1.0 (0.85)	-1.999	0.046	-0.10

Likelihood ratio Chi-square-test or Mann-Whitney U-test used to compare the groups.

No significant differences existed between the adolescents and adults in PCL-R total scores, factor 2 (social deviance) scores, or in facets 3 (lifestyle) and 4 (antisocial). Adults scored significantly higher on factor 1 (interpersonal/affective) and facets 1 (interpersonal) and 2 (affective). No significant correlation existed between offender's age and PCL-R in adolescents. A negative correlation between offender's age and PCL-R total score in the comparison group was observed (Pearson r = -0.323, p = 0.014).

The adolescent sample was divided into two groups according to PCL-R total scores. 45 boys scored less than 26 points and 12 boys scored 26 or more points on the PCL-R. In order to analyse whether the two groups differ in terms of all of the PCL-R factors and facets, Table 2 presents the mean PCL-R scores for both groups. In addition, results are presented for the two groups with regard to diagnoses, crime scene behavior, victim-offender relationship, crime history, and life course development.

**Table 2:** Mean (SD) Factors, facets, clinical diagnoses, crime history, crime characteristics, victim-offender relationship, and life course development in homicidal male adolescents scoring low (total score < 26; n= 45) and high (total score ≥ 26; n= 12) on the PCL-R.

	PCL-R < 26	PCL-R ≥ 26	Statistics	p	Phi
*Psychopathy*					
PCL-R factor 1 Interpersonal/Affective	4.07 (2.38)	10.7 (1.93)	-5.065	0.000	-0.78
PCL-R factor 2 Social Deviance	9.8 (5.03)	17.42 (1.73)	-4.064	0.000	-0.62
PCL-R facet 1 Interpersonal	0.6 (0.83)	3.9 (1.47)	-5.279	0.000	-0.81
PCL-R facet 2 Affective	3.93 (2.83)	6.75 (1.05)	-3.970	0.001	-0.61
PCL-R facet 3 Lifestyle	5.74 (3.48)	9.52 (0.71)	-3.320	0.001	-0.51
PCL-R facet 4 Antisocial	4.41 (2.34)	8.25 (1.61)	-4.235	0.000	-0.65
					
*History of previous offending*					
Previous crime history	19/45 (42%)	9/12 (66%)	4.073	0.044	0.27
Violent crime (assault, homicide, rape)	12/44 (27%)	5/12 (42%)	0.924	0.336	0.13
Property crime	16/44 (36%)	8/12 (67%)	3.535	0.060	0.25
Drug-related	3/44 (7%)	2/12 (17%)	†	0.619	0.10
					
*Clinical diagnoses during the index homicide*					
Psychotic disorder	2/45 (4%)	2/12 (17%)	†	0.141	0.20
Organic disorder	1/45 (2%)	1/12 (8%)	†	0.380	0.14
Depressive disorder	9/45 (20%)	4/12 (19%)	†	0.440	0.13
Conduct disorder or anti-social personality disorder	20/45 (44%)	9/12 (75%)	3.539	0.060	0.25
Another than antisocial personality disorder	21/45 (47%)	9/12 (75%)	3.051	0.081	0.23
Alcohol abuse/dependence	20/45 (44%)	7/12 (58%)	0.733	0.392	0.11
Substance abuse	11/45 (24%)	6/12 (50%)	2.956	0.086	0.23
No diagnosis	3/45 (7%)	0/12 (0%)	†	0.358	-0.12
					
*Crime characteristics*					
Excessive violence during the index homicide	4/44 (9%)	4/11 (36%)	†	0.042	0.31
More than one offender	12/41 (29%)	7/12 (58%)	3.410	0.065	0.25
More than one victim	3/41 (7%)	0/12 (0%)	†	0.335	-0.13
Another crime in association with the homicide	18/40 (45%)	8/11 (73%)	2.654	0.103	0.23
					
*Victim-offender relationship*					
Family	9/44 (21%)	1/11 (9%)	†	0.667	-0.12
(Ex-)intimate	1/44 (2%)	1/11 (9%)	†	0.363	0.15
Acquaintance	24/44 (55%)	7/11 (64%)	0.296	0.587	0.07
Stranger	10/44 (23%)	2/11 (18%)	†	1.000	0.04
					
*Life course development*					
Lived with both parents until 16 years of age	22/45 (49%)	1/12 (8%)	†	0.018	-0.34
Institutional or foster home placement in childhood	14/45 (31%)	7/12 (58%)	2.387	0.029	0.29
Parental alcohol abuse	26/44 (59%)	8/10 (80%)	1.528	0.216	0.17
Parental psychiatric problems	7/41 (17%)	4/11 (36%)	†	0.164	0.19
Physical violence at childhood home	15/45 (33%)	6/11 (55%)	1.697	0.193	0.17
Parental criminal history	8/44 (18%)	7/12 (58%)	7.751	0.005	0.37
Homicide history of parents or near relatives	8/44 (18%)	7/12 (58%)	7.751	0.005	0.37
School difficulties	31/45 (69%)	12/12 (100%)	4.949	0.026	0.30
Special education	18/45 (40%)	12/12 (100%)	13.680	0.000	0.49
Mental health contact prior to age 18 years	19/45 (42%)	9/12 (75%)	4.073	0.044	0.27

Likelihood ratio Chi-square-test or Fisher's two-sided exact test (†) used to compare the groups.

The boys scoring high on the PCL-R differed markedly from those scoring low on the PCL-R on both factors and all four facets. They significantly more often had a crime history before the index homicide, more frequently used excessive violence during the index homicide, more rarely lived with both parents until 16 years of age, had more institutional or foster home placements in childhood, had more school difficulties, more often had received special education, and, more often had contact with mental health services prior to age 18 years than boys scoring low on the PCL-R. They also more often had parental criminal history as well as homicide history of parents or near relatives than the group scoring low on the PCL-R.

## Discussion

The aim of this study was to evaluate psychopathy-like personality traits in a nationwide consecutive sample of adolescent male homicide offenders and to compare these findings with a randomly sampled adult gender-matched homicide offender group. According to our hypothesis PCL-R total scores did not differ between the two groups. Homicidal boys as a group displayed highly antisocial behavior, and despite being approximately 20 years younger than the adult offenders, were as antisocial as the adult group. The adults, however, showed more both affective and interpersonal features of psychopathy. Many authors contend that psychopathic personality features – such as dishonesty, lack of guilt, or manipulativeness – are often present already in childhood [9,10,66,67], but interpersonal and affective features typically develop and strengthen over time in connection with other people [67]. This might explain why they were not as prominent in youths, with fairly limited social interaction networks, as in adults. In addition, this may explain the recent reports that young offenders with psychopathy-like personality character might be more malleable than adults and benefit more from treatment [68]. Another explanation for the difference could be that the affective-interpersonal features were similarly present in adolescents and adults, but were not noticed equally well by forensic investigators, who typically work in the field of adult psychiatry and are not experts in adolescent psychiatry. It is also noteworthy that in Finland approximately only one-tenth of forensic psychiatric examinations are performed on under-aged offenders. The affective-interpersonal aspects of psychopathy are typically more difficult to recognize than the behavioral ones and the difficulty may be even greater in youngsters. To better understand the transition from psychopathic features to adult psychopathy, more prospective longitudinal research is needed.

In line with the previous Finnish study [7], the mean age of the victims did not differ between the adolescents and the adult comparison group. This has been explained by two main reasons. Firstly, parents, stepparents as well as other adult quardians are often victims of under-aged homicide offenders, and, secondly, robbery is one of the most frequent motives among adolescent killers [6]. Among Finnish adults, a typical homicide victim is an adult drinking partner [5]. The other aim of the present study was to investigate associations between adolescent psychopathic traits and offender and offence characteristics. The adolescent sample was divided into two groups according to PCL-R total scores. Approximately one in five Finnish homicidal male adolescents showed psychopathy-like personality character using the PCL-R with the recommended cut-off score of 26 for Scandinavian populations. There is continuous ongoing discussion on whether antisocial and criminal behaviors are parts of the psychopathy syndrome. In a recent study by Andershed et al. [69], the three-factor model of psychopathy by Cooke and Michie [70], which excludes aspects of criminal behavior, was shown to be useful in identifying a problematic subgroup of young offenders. In the present study, boys with psychopathy-like personality character differed markedly from those scoring low on the PCL-R on both factors and all four facets. Thus, differences between the groups cannot be explained only by differences in antisocial and criminal behaviors but also by both affective and interpersonal features, which are often known as the "core" of psychopathy [9]. Many studies among children and adolescents indicate that these affective-interpersonal traits foreshadow the greatest risk of long-term maladjustment [71-73].

Boys with psychopathy-like personality character significantly more often had a previous crime history than boys scoring low on the PCL-R. This result is in agreement with many previous reports showing that adolescents with psychopathic traits differ from other antisocial youths in age of onset of criminal career and likelihood of recidivism [33-35,74,75].

In line with previous studies on Finnish adolescent homicide offenders, our results suggest that abuse of alcohol or drugs, personality disorders, and social maladjustment characterize young offenders [6,7]. In accordance with the results for adult populations and for some earlier studies among young offenders [41,70,76], adolescents scoring high on the PCL-R seemed to have a tendency to suffer from conduct disorder/antisocial personality disorder as well as from other personality disorders and substance abuse/dependence more often than adolescents scoring low on the PCL-R.

The degree of cruelty in a homicide is difficult to measure, and a finding of brutality is subjective, varying across time and place. In our study, the use of excessive violence in killing was significantly more frequent in boys with psychopathy-like personality character than in boys scoring low on the PCL-R. Despite our conservative criteria for excessive violence, almost 40% of the boys scoring high on the PCL-R were classified into this category. The finding is similar to that reported in adult populations and is consistent with the study by Murrie et al. [77], in which PCL-YV psychopathy scores correlated with measures of severity of violence among juvenile male offenders. Also, in the recent study by Kruh et al. [78], affective-interpersonal features of psychopathy-like personality character predicted more frequent use of sadistic violence, repeated violence against the same victim, and violence resulting in more serious victim injuries.

Adolescents with either childhood-onset or adolescent-onset conduct disorder have equally high levels of delinquent peer affiliation [79], but youngsters with both conduct problems and affective-interpersonal features of psychopathy-like personality character show the highest level of affiliation with deviant peers [80]. In line with these earlier findings, a tendency was observed that boys scoring high on the PCL-R more often committed a homicide together with one or more co-offenders than boys scoring low on the PCL-R.

In accord with previous studies among adolescent homicide offenders, acts of instrumental violence were very common. In line with a study by Kruh et al. [78] there was some positive association between instrumental violence and psychopathy-like personality character but it failed to reach a statistically significant level and the effect size was only moderate.

One of the aims of the study was to examine connections between adolescent psychopathic traits and life course development because adverse family conditions and early traumatization are commonly regarded as risk factors for aggressive and violent behavior [81,82], and high victimization subjects have been reported to have high PCL-R scores in adulthood [25]. In our study, boys with high psychopathy-like personality character more rarely lived with both parents until the age of 16 years and had more institutional or foster home placements in childhood than boys scoring low on the PCL-R. This is in line with a recent study by Campbell et al. [26], in which a history of nonparental living arrangements (e.g. foster care) predicted higher PCL-YV scores among a sample of incarcerated adolescent offenders. Correlations between high PCL-R scores and broken homes as well as between psychopathy and single-parent families have also been reported earlier [83,84]. In line with previous studies [27,28,85], there was some positive association between parental alcohol abuse, mental health problems, physical abuse and psychopathy-like personality character but it failed to reach a statistically significant level and the effect size was only moderate. Although the quality of parenting generally predicts externalizing behavior, according to some recent research [85-87], ineffective parenting might be less relevant in explaining behavioral problems of children with callous-unemotional traits of psychopathy-like personality character. The connection between parental history of antisocial behavior and child conduct problems has been well documented [88,89]. Recent studies have also revealed strong evidence of inter-generational transmission of criminality from parents to offsprings [90,91]. Here, boys with psychopathy-like personality character significantly more often had parental criminal history as well as homicide history of parents or near relatives than boys scoring low on the PCL-R. The finding is in accordance with Christian et al. [92] and Frick et al. [93], who found that children with conduct problems and features of psychopathy were more likely to have parents with a history of antisocial personality disorder as well as fathers with an arrest history than children with conduct problems only.

Severe antisocial behavior has often been demonstrated to be associated with low intelligence [94,95]. Recent studies, however, suggest that youths with psychopathic traits do not show the verbal intelligence deficits generally associated with conduct problems, and the relationships between psychopathic features and nonverbal intelligence have not been clearly identified [34]. In our study, all adolescent homicidal offenders scoring high on the PCL-R had experienced school difficulties and received special education, and in this sense they differed significantly from boys scoring low on the PCL-R. Both school difficulties and the need for special education are typically based on either medical, psychological, social, or intellectual factors, but we were unable to specify individual reasons. The issue is clearly important and warrants future research.

Adult psychopathy is often described as incurable syndrome [96], and from the perspective of adult psychiatry, it would be important and cost-effective to identify those at risk of psychopathic behavior as early as possible. In our study, up to 75% of the boys with psychopathy-like personality character had had some contact with mental health services – via either child or adolescent psychiatry – prior to the index homicide. Better recognition of childhood trauma and other characteristics of youngsters with psychopathic traits would facilitate effective prevention and intervention efforts. The families with risk factors need extensively help and the children protection for avoiding the "cycle of adverse experiences" as well as the "cycle of violence" [97]. In addition, there is some new evidence that young offenders with psychopathy-like personality character might be more malleable than adults and benefit more from treatment [68].

A strength of this study was its nationwide comprehensive nature. The high Finnish clearance rate for homicide, the tradition of thorough forensic psychiatric examinations, and reliable statistics form a solid basis for register-based study. However, the fact that the present study was retrospective and register-based does present some obvious limitations, though the same limitations apply both the adolescents and the comparison group. Unfortunately, it was not possible to estimate the representativeness of the whole sample by comparing the number of offenders in the present sample with the number of overall adolescent homicide. In a previous Finnish study [7], it was estimated that 60% of 15–17-year old homicide suspects go through the forensic examination. The selection bias applying both the adolescents and the comparison group is, however, a clear limitation of the study, and must be kept in mind. In addition, regardless of the fact that the adolescent sample consisted of all homicidal boys who underwent forensic psychiatric examinations and were convicted in 1995–2004, the number of subjects remained small and the results must be regarded as indicative. One must also remember that the concept of psychopathy is not an established medical diagnosis, and its applicability to emotionally immature adolescents from deprived backgrounds serves further studies. Also, the observed effect sizes were not large, with phi's falling mostly into category of moderate effect size [64]. Finally, due to multiple comparisons, there is a possibility that statistical significance was declared where no association exists (type I error).

## Conclusion

Homicidal boys behaved as antisocially as the homicidal adults. The adults, however, showed more both affective and interpersonal features of psychopathy. Homicidal adolescents with psychopathy-like personality character form a special subgroup among other homicidal youngsters. Recognizing their characteristics, especially in life course development, would facilitate effective prevention and intervention efforts.

## Competing interests

NL has received travel funds from Novartis and NL and HH-N from BMS during 2008.

The other authors declare no competing interests.

## Authors' contributions

NL reviewed the forensic psychiatric statements, scored for the PCL-R, organized data, and served as the first author. TL analyzed data. MH participated in the writing process. HP participated in the writing process. GW-H reviewed the forensic psychiatric statements, scored the PCL-R, and participated in the writing process. HH-N provided the material, contributed with ideas and context, and participated in the writing process.

## Pre-publication history

The pre-publication history for this paper can be accessed here:


